# A study on the measurement of GSR with bloodstains by ICP-MS

**DOI:** 10.1093/fsr/owad033

**Published:** 2023-11-29

**Authors:** Xiang Li, Aoyang Yü, Xinxin Xia, Yü Zhu, Hui Song

**Affiliations:** Department of Forensic Chemistry, Criminal Investigation Police University of China, Shenyang, China; Department of Trace Examination, Criminal Investigation Police University of China, Shenyang, China; Department of Forensic Chemistry, Criminal Investigation Police University of China, Shenyang, China; Department of Forensic Chemistry, Criminal Investigation Police University of China, Shenyang, China; Department of Forensic Chemistry, Criminal Investigation Police University of China, Shenyang, China

**Keywords:** forensic science, gunshot residues, bloodstains, ICP-MS, hand, cloth

## Abstract

In forensic laboratories, analytical investigations of gunshot residues (GSRs) are usually conducted by scanning electron microscopy (SEM) in combination with energy dispersive X-ray microanalysis. If GSRs are covered with bloodstains, SEM cannot detect them. In this study, an inductively coupled plasma mass spectrometry method is proposed to solve this problem. Results show that bloodstains did not interfere with GSRs and low-level elements are detected. Qualitative and quantitative analyses of Sn, Sb, Ba, and Pb elements in GSRs are also carried out. Different pretreatment methods are adopted according to the characteristics of different samples. Our investigations suggest that the proposed method has the advantages of low detection limit and high sensitivity and it can be very important in expert testimony.

**Key points:**

- GSRs with bloodstains could be successfully detected *via* ICP-MS and bloodstains did not interfere in GSRs analysis.
- The best pretreatment method for incident bullet holes with bloodstains was microwave digestion.
- The best pretreatment method for the region around the bullet hole and the shooter’s hand with bloodstains was ultrasonic vibration.
- For the same shooting distance, GSRs collected from the cloth and the shooter’s hand with type 95-1 rifle were higher than that of type 92 pistol.

## Introduction

Powerful lethality of firearms not only causes serious injury to people but also poses a substantial psychological threat to others, causing significant harm to social and public security [1]. China has always adhered to the policy of strict control of guns and considered the curbing of gun-involved cases as an important task to safeguard national security, social security, and stability [1]. Type 95-1 rifle is also referred to as QBZ-95-1. QBZ is a class code derived from the first letter of Chinese Pinyin for “light arms-rifles-automatic”. Type 92 pistol has been developed to replace the originally equipped type 54 pistol and, is available in 5.8 and 9 mm calibres. At present, the type 92 pistol used for police distribution is mainly of 9 mm calibre. Type 92 pistol and type 95-1 rifle are the most widely used guns in China.

The formation and distribution of gunshot residues (GSRs) are closely related to the target and firing distance. GSRs on the shooter’s body and hand are caused by the destruction of seal between the cartridge and the bore wall during the cartridge throwing process. The gas–solid flow containing explosive and projectile residues leak from the joint of the ejector port, sleeve, and gun body. GSRs on the object are ejected with residual particles as the projectile leaves the muzzle. At close range, GSRs attach to the impact point and forms a powder fog [2–4].

Sn, Sb, Ba, and Pb are the four most commonly used elements in testing shooting residues. Sn originates from the tinfoil cover in the primer, whilst Sb derives from antimony sulphide (Sb_2_S_3_) which is used as a combustible agent. Ba originates from barium nitrate (Ba(NO_3_)_2_), used as the oxidant, and Pb is derived from lead styphnate (C_6_HN_3_O_8_Pb) which is the primary explosive. The detection of gun-involved cases often requires the identification of the body part of the wounded personnel, ballistic trajectory, shotgun, and so on. Forensic chemists need to analyse the inorganic elements in GSRs to provide scientific evidence for charges in court. Inductively coupled plasma mass spectrometry (ICP-MS) is an inorganic element analysis technique that uses inductively coupled plasma as the ion source and a mass spectrometer as the detector. In forensic analysis, many studies have been published to date to demonstrate the feasibility of ICP-MS for the identification of metallic elements in GSRs [5–10].

A colorimetric test is generally used to investigate GSRs [11] due to its simple and quick operation. However, bloodstains affect the colour reaction. Thus, it is necessary to find clear metal particles [12–16] when examining GSRs *via* scanning electron microscopy (SEM) in combination with energy dispersive X-ray (EDX) microanalysis. Polovkova et al. [17] conducted SEM–EDX investigations to find that bullet manufacturers add different metal elements to different types of bullets to mark the type of ammunition. They further found that for GSRs covered in bloodstains, it was not easy to identify the metal particles. Panarin et al. [18] studied the application of Raman spectroscopy for the detection of GSRs and showed that the method exhibited a high sensitivity to metal elements, which provided a new research direction for the detection of GSRs *via* Raman spectroscopy [19, 20], and the proposed method was susceptible to the environment. Wunnapuk et al. [21] proposed an ICP-MS based method to study GSRs and found that the elemental composition of GSRs originated from full-jacketed bullets and that from lead bullets were significantly different. It was later shown that ICP-MS could analyse trace elements in GSRs [5–10]. Steffen [10] proposed a combination of ICP-MS and SEM–EDX for these investigations. They obtained good results, but the proposed methodology was time-consuming. Comparing all these approaches, ICP-MS provides an accurate quantitative analysis which high sensitivity and good reproducibility.

At present, Sn, Sb, Ba, and Pb elements in GSRs are usually examined by SEM–EDX. However, it is difficult to examine the shooting residues on clothes and hands, wrapped in bloodstains. SEM cannot find GSRs dissolved in the blood. Therefore, an ICP-MS based method is proposed in this research which can solve this problem. GSRs are quantitatively analysed by ICP-MS and semi-quantitatively analysed by SEM. The distribution of GSRs is studied by ICP-MS. ICP-MS analysed GSRs around the incident bullet hole, at the bullet hole, and on the hand with bloodstains. The proposed method is simple, rapid, and efficient. Obtained results suggest that this approach does not interfere with bloodstains and can thus be used to detect GSRs in different samples.

## Experimental methods

### Materials and reagents

**Figure 1:**
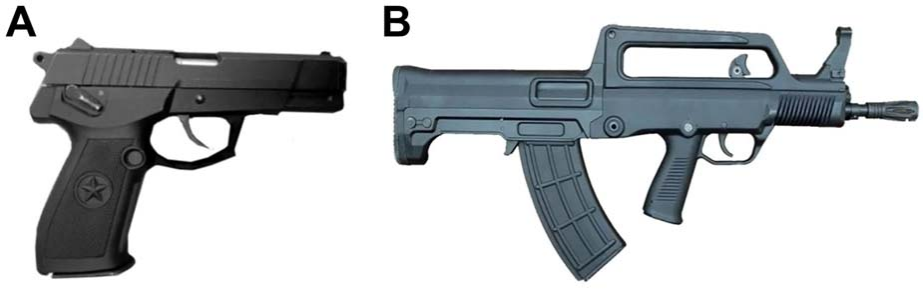
Photographs of a type 92 pistol (A) and a type 95-1 rifle (B).

Samples were provided by the ballistics laboratory in the Criminal Investigation Police University of China. Firearms used in the experiment were a type 92 pistol (Calibre, 9 mm; Cartridge, DAP10A) and a type 95-1 rifle (Calibre, 5.8 mm; Cartridge, DAP10A) (Figure 1).

Woven polyamide fabric (0.01 g/cm^2^) served as the target material. The fluid pig blood was obtained from the butchery during professional slaughtering.

Ultrapure 65% nitric acid (HNO_3_) (Sinopharm, Beijing, China) and ultrapure water (18.2 MΩ cm) obtained from a reverse osmosis system (Millipore S.A.S., Molsheim, France) were used for sample preparation. The cloth material was dacron. A multi-element solution (1 000 mg L^−1^) for Sn, Sb, Ba, and Pb standards (Guobiao Testing & Certification, China) was prepared to establish the calibration curve (10, 20, 30, 50, 80, and 100 μg/L). All standard solutions were diluted with 2% HNO_3_.

### Equipment

An ICP-MS (iCAP Q; Thermo Fisher, West Palm Beach, FL, USA) was used for the quantification of Sn, Sb, Ba, and Pb elements. The operating parameters listed in Table 1 were optimised using a quality calibration and automatic tuning system. Samples were extracted in an ultrasonic bath (KQ-200VDE; Kunshan Shumei, Shanghai, China).

**Table 1:** Operation conditions for Inductively coupled plasma mass spectrometry (ICP-MS) instrument

Item	Operation conditions
RF power	1 553.2 W
Detector voltage	927 V
Nebuliser (Ar)	1.05 L/min
Coolant (Ar)	13.72 L/min
Auxiliary	0.791 L/min
Sampling depth	5.10 mm
Take-up time	30 s
Wash time	30 s
Replicate	3

### Experimental procedures

#### Collection of samples

To evaluate the best collecting method on hands with bloodstains, samples from the thumb and index finger of the volunteer were collected before shooting as blank samples, and samples of blank swabs with bloodstains were also collected. The GSR was acquired from seven male volunteers (six with type 92 pistol and one with type 95-1 rifle). After the volunteer shot with the type 92 pistol, 100 μL of blood was placed on the thumb and index finger, and then wiped with a swab. Samples were collected after the volunteer washed his/her hands once, twice, and three times. Six samples (blank hand, blank swab with bloodstains, 1 shot, 1 shot and 1 washing, 1 shot, and 2 washing, and 1 shot and 3 washing) were collected for each extraction method, and 30 samples for five different extraction methods were collected.

**Figure 2:**
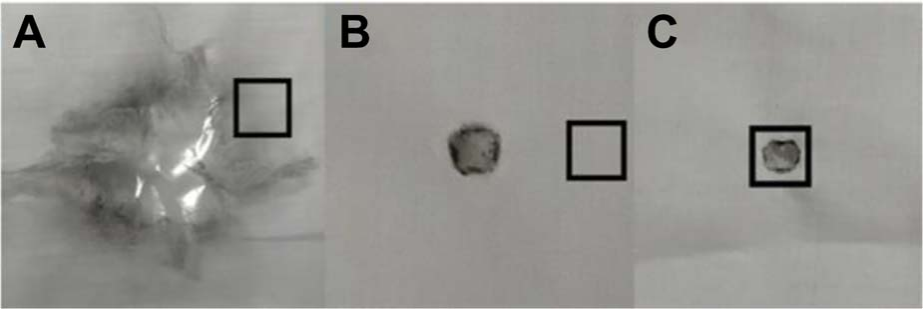
Samples (1 cm × 1 cm) around the incident bullet hole (A,B) and at the centre of incident bullet hole (C).

Two pretreatment methods including microwave digestion and ultrasonic vibration were used for the cloth with bloodstains. GSRs were deposited on the surface of the white cloth using type 92 pistol and type 95-1 rifle. Samples (1 cm × 1 cm) with bloodstains were taken at a distance of 1.5, 2.5, and 3.5 cm from the bullet hole for a made by a type 92 pistol for a 2 cm shooting distance (Figure 2). Samples and pretreatment methods for the type 95-1 rifle and type 92 pistol were the same. Each experiment was repeated three times and 36 samples were collected.

The extraction effect of microwave digestion and ultrasonic vibration methods on the incident bullet holes with bloodstains was investigated (Figure 2). Since the incident bullet hole in the cloth would be damaged when shooting at a close range, the shooting distance was kept at 60 and 160 cm. Six samples with different shooting distances were collected.

The distance between the shooter and target was set at 20 and 60 cm. Considering that the cloth was torn during close shooting, samples from a different shooting distance were taken at 1.5 cm from the centre of the incident bullet hole. Surgical blades were used to cut the cloth and were replaced each time. A total of 100 μL blood was dripped onto a 1 cm × 1 cm cloth. After drying the blood, the cloth was used for testing. Data were collected three times for each distance, and 12 samples were collected.

One volunteer shot with type 92 pistol, while a second volunteer shot with type 95-1 rifle. Before shooting, samples from the thumb and index finger were collected as blank samples. After volunteers shot with different firearms, 100 μL of blood was placed on the thumb and index finger, and then wiped with a swab. After one shot, the volunteers washed their hands once, twice, and three times and samples were collected after each wash. The volunteers washed their hands following a similar procedure, namely, using soap and deionised water and, giving a total of 12 samples.

#### Microwave digestion

A total of 6 mL of HNO_3_ and 2 mL of 30% H_2_O_2_ were placed in PTFE jars, and the cloth with bloodstains was added. The sample was left standing for 1 h and digested by microwave digestion. Experimental conditions are shown in Table 2. The digestion solution was evaporated at 150°C to obtain a 0.5 mL sample. After cooling, the solution was diluted to 10 mL.

**Table 2:** Microwave digestion conditions.

Parameters	Step 1	Step 2	Step 3
Temperature (°C)	120	150	190
Time (min)	5	10	15
Power (W)	900	900	900

#### Ultrasonic vibration

A total of 2 mL of 10% HNO_3_ (*v*/*v*) was placed in 15 mL polypropylene tubes and the cloth with bloodstains was added to the tube. Samples were placed in an ultrasonic bath at 45 kHz and 80°C for 30 min. Ultrapure water (8 mL) was added to tubes and the solution volume was kept at 10 mL.

### Statistical analysis

Concentrations (μg/L) of Sn, Sb, Ba, and Pb were obtained from the ICP-MS analysis of samples solutions. Statistical analyses were performed by using Microsoft Excel 2010 (Microsoft Co., Redmond, WA, USA). Pearson’s correlation was used to investigate the correlation between parameters at a 95% confidence interval. The significance level was set to 0.05. Differences in element contents between microwave digestion and ultrasonic vibration of pretreatment at bullet entrance hole were compared using a Student’s *t*-test. Differences in element contents between fabric samples with and without bloodstains were also compared with Student’s *t*-test. Data were presented as the mean ± standard error.

## Results and discussion

### Method evaluation

#### GSRs on cloth with bloodstains

The analytical curve was produced with the concentrations of 0.5, 10, 30, 50, 80, and 100 μg/L. The limit of detection (LOD) and limit of quantitation (LOQ) were 3 and 10 times the standard deviation of signals generated by 11 tests of blank cotton, respectively. The accuracy of the method was verified by recovery of blank addition, calculated according to the following formula:


(1) \begin{equation*} \text{Recovery}=\left\{\left(\left[\ \right]\text{blank}+\text{addition}-\left[\ \right]\text{blank}\right)/\left[\ \right]\text{standard}\right\}\times 100. \end{equation*}

Table 3 shows the LOD, LOQ, recovery, and *R*^2^ values obtained from the ICP-MS analysis on cloth with bloodstains using the ultrasonic vibration pretreatment method. The corresponding values using a microwave digestion method were reported elsewhere [20].

**Table 3:** Values obtained from ICP-MS analysis on cloth with bloodstains by ultrasonic vibration.

Isotope	LOD (μg/L)	LOQ (μg/L)	Recovery (%)	*R* ^2^
Sn	1.04	3.43	91–115	0.9991
Sb	0.97	3.21	81–89	0.9994
Ba	1.68	5.54	92–113	0.9993
Pb	0.78	2.57	93–106	0.9994

LOD: limit of detection; LOQ: limit of quantification.

Table 4 shows the LOD, LOQ, recovery, and *R*^2^ values obtained from the ICP-MS analysis performed on swabs with bloodstains using the ultrasonic vibration pretreatment method.

**Table 4:** Values obtained from Inductively coupled plasma mass spectrometry (ICP-MS) analysis on swab with bloodstains by ultrasonic vibration.

Isotope	LOD (μg/L)	LOQ (μg/L)	Recovery (%)	*R* ^2^
Sn	0.85	2.81	92–110	0.9992
Sb	0.93	3.70	82–91	0.9991
Ba	1.56	5.15	93–111	0.9992
Pb	1.24	4.09	94–107	0.9995

LOD: limit of detection; LOQ: limit of quantification.

### Selection of experimental conditions

#### Determination of the best collection method on hands with bloodstains

To find the best method for collecting GSR from hands with bloodstains, water, 2% EDTA, 0.2% nitric acid, 2% nitric acid, and 10% nitric acid were used to collect GSR from the thumb and index finger of a volunteer shooter using a type 92 pistol. According to a previous report [20], the thumb and index finger of the shooter were the best areas for collection. The best collection method on hands with bloodstains was investigated by collecting samples on the thumb and index finger.

Table 5 shows the Sn, Sb, Ba, and Pb concentrations of GSRs using different collecting methods. Swabs moistened with 2% EDTA solution (*w*/*v*) exhibited the high sensitivity to the collection of GSRs, and Sn, Sb, Ba, and Pb elements were extracted from the blood. The effect of different concentrations of HNO_3_ solution (*v*/*v*) including 0.2%, 2%, and 10% was investigated in the experiment. Theoretically, with increasing HNO_3_ concentration, more GSRs were dissolved in HNO_3_ solution, and more GSRs were extracted. The Sb (78.84 μg/L) content decreased significantly when a 0.2% HNO_3_ solution (*v*/*v*) swab was used to collect samples. A total of 10% HNO_3_ solution (*v*/*v*) was corrosive to the skin. Swabs moistened with 2% HNO_3_ solution (*v*/*v*) were selected for collecting GSR with bloodstains on the shooter’s hand.

**Table 5:** Concentrations (μg/L) of Sn, Sb, Ba, and Pb in GSRs were obtained by different collection methods for a single shot and different numbers of hand washes (one, two, and three).

Samples	Sn (μg/L)	Sb (μg/L)	Ba (μg/L)	Pb (μg/L)
Swab moistened with water				
Blank hand	<LOD	2.31	34.27	11.32
Blank swab with bloodstains	2.15	<LOD	5.42	<LOD
1 shot	343.58	82.44	126.56	228.16
1 shot and 1 washing	36.51	31.21	42.51	52.14
1 shot and 2 washing	31.52	26.58	55.24	35.26
1 shot and 3 washing	2.45	7.06	36.84	13.56
Swab moistened with EDTA solution (2% *W/V*)				
Blank hand	<LOD	1.62	39.56	17.67
Blank swab with bloodstains	6.25	<LOD	8.01	1.94
1 shot	337.51	105.42	152.41	262.45
1 shot and 1 washing	123.25	55.26	152.58	125.24
1 shot and 2 washing	21.53	35.36	64.28	42.56
1 shot and 3 washing	6.35	13.56	42.15	25.12
Swab moistened with HNO_3_ solution (0.2% *W/V*)				
Blank hand	1.87	3.51	31.58	18.19
Blank swab with bloodstains	4.04	<LOD	6.59	2.45
1 shot	521.37	78.84	213.25	345.26
1 shot and 1 washing	167.8	64.58	12.4	90.26
1 shot and 2 washing	11.21	26.45	52.64	31.26
1 shot and 3 washing	8.51	19.52	57.26	25.86
Swab moistened with HNO_3_ solution (2% *W/V*)				
Blank hand	3.18	1.336	25.81	21.98
Blank swab with bloodstains	2.71	<LOD	2.41	1.26
1 shot	491.82	134.25	156.22	311.65
1 shot and 1 washing	152.25	72.56	106.25	89.64
1 shot and 2 washing	31.52	15.26	39.24	30.25
1 shot and 3 washing	14.31	9.54	42.52	29.53
Swab moistened with HNO_3_ solution (10% *W/V*)				
Blank hand	1.78	3.05	30.87	27.45
Blank swab with bloodstains	1.56	<LOD	1.82	<LOD
1 shot	462.14	112.8	124.28	326.21
1 shot and 1 washing	134.21	52.14	95.52	76.85
1 shot and 2 washing	16.25	21.58	39.85	25.26
1 shot and 3 washing	7.41	14.25	36.2	20.73

LOD: limit of detection.

**Figure 3:**
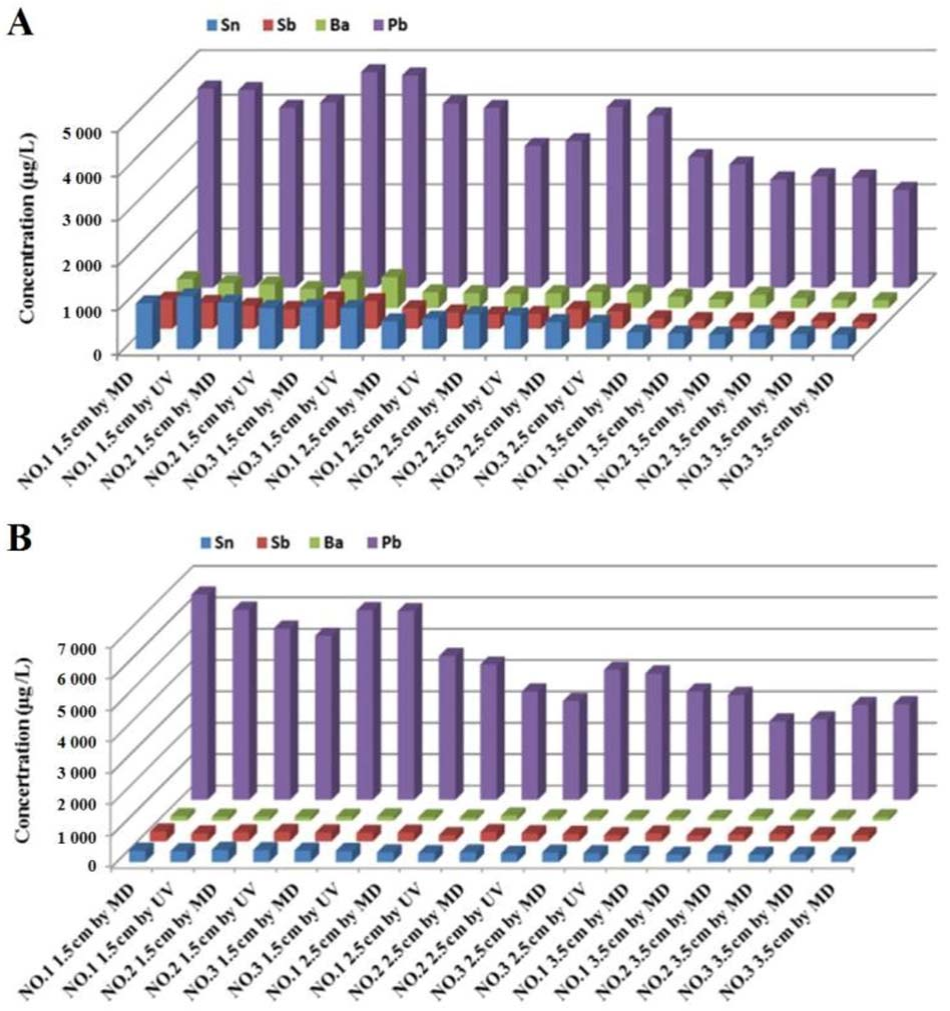
Concentrations of Sn, Sb, Ba, and Pb in GSRs from type 92 pistol (A) and type 95-1 rifle (B) on cloth with bloodstains by microwave digestion (MD) and ultrasonic vibration (UV).

#### Determination of the best pretreatment method on cloth with bloodstains

**Figure 4:**
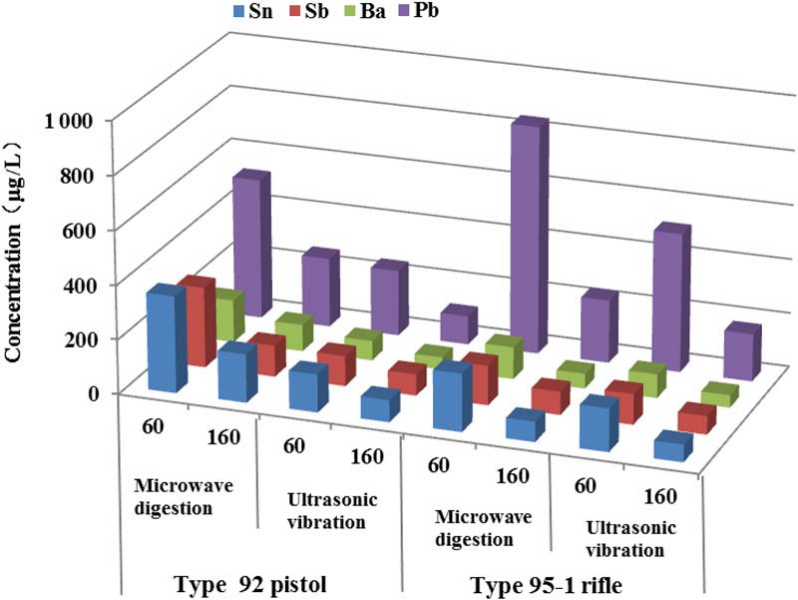
Concentrations of Sn, Sb, Ba, and Pb in GSRs obtained by microwave digestion and ultrasonic vibration on incident bullet holes with bloodstains at shooting distances of 60 and 160 cm.

To find the best pretreatment method for cloth with bloodstains, concentrations of Sn, Sb, Ba, and Pb in the GSRs were determined for type 92 pistol (Figure 3A) and type 95-1 rifle (Figure 3B). Figures 3B and 4 show that the concentrations of Sn, Sb, Ba, and Pb in shooting residues for the same guns in the same regions were similar between microwave digestion and ultrasonic vibration treatment. The data show that the concentrations of Sn, Sb, Ba, and Pb in samples treated *via* microwave digestion were slightly higher than those treated by ultrasonic vibration.

**Table 6:** Element contents (μg/L) in cloth with and without bloodstains.

Sample	Sn (μg/L)		Sb (μg/L)		Ba (μg/L)		Pb (μg/L)	
	W	WO	W	WO	W	WO	W	WO
1	96.25	93.26	82.64	87.65	106.84	102.47	95.37	105.47
2	94.27	101.21	89.37	84.49	110.48	109.52	97.26	94.73
3	95.22	92.63	82.84	89.83	96.59	96.73	103.85	98.47
4	103.42	95.47	86.93	86.48	98.57	110.65	94.97	105.64
5	93.46	96.78	90.38	85.48	105.48	98.69	96.84	106.45
6	96.58	94.67	83.42	87.57	107.56	103.67	105.47	97.38
Mean	96.53	95.67	85.93	86.92	104.25	103.62	98.96	101.36

W: with bloodstains; WO: without bloodstains.

**Table 7:** Element contents (μg/L) in swab with and without bloodstains.

Sample	Sn (μg/L)		Sb (μg/L)		Ba (μg/L)		Pb (μg/L)	
	W	WO	W	WO	W	WO	W	WO
1	92.45	95.62	90.26	89.52	93.45	98.56	98.82	95.29
2	98.27	97.52	85.36	85.26	97.52	94.52	95.37	94.26
3	94.36	102.84	87.25	89.62	106.25	96.25	94.25	102.75
4	97.52	93.67	82.15	90.73	98.15	94.56	96.52	95.64
5	101.85	94.56	81.27	82.12	109.53	108.26	105.37	94.28
6	105.37	95.38	89.25	93.62	110.61	110.68	103.75	105.38
Mean	98.30	96.60	90.67	88.48	102.59	100.47	99.01	97.93

W: with bloodstains; WO: without bloodstains.

The digesting container was closed for microwave digestion. Both the material and reagent did not have a volatile loss, and the process was easily automated. Ultrasonic vibration was used to extract the components directly from the sample, without completely decomposing the organic matter. Furthermore, this method was not affected by the intrinsic impurities of the cloth and bloodstains. This method required the transfer of components to be measured in the solution, thus the test dose was relatively small. The treatment process was simple and the conditions were non-destructive. Additionally, the blank value was low, and the possibility of loss or contamination of components was small. The ultrasonic vibration method was more suitable to analyse GSRs of cloth with bloodstains.

To compare the best pretreatment method for cloth on incident bullet holes with bloodstains at shooting distances of 60 and 160 cm, average concentrations of Sn, Sb, Ba, and Pb in the GSRs were determined. Results in Figure 4 show that, for the same gun with the same shooting distance, the extraction efficiency of the microwave digestion method (358.12, 297.36, 157.28, and 521.28 μg/L for Sn, Sb, Ba, and Pb with type 92 pistol at 60 cm) on the incident bullet holes was superior to that obtained with the ultrasonic vibration approach (139.62, 111.84, 72.79, and 243.46 μg/L for Sn, Sb, Ba, and Pb with type 92 pistol at 60 cm). The extraction of GSRs around the bullet hole had a similar effect (data not shown).

### Investigation of the influence of bloodstains on GSRs

It is difficult to find two samples with identical Sn, Sb, Ba, and Pb contents in GSRs after shooting. The standard addition method for Sn, Sb, Ba, and Pb in the blood was used to investigate the influence of bloodstains on GSRs. A total of 10 μL of 100 μg/mL Sn, Sb, Ba, and Pb standards and 100 μL blood was added to cloth and swab samples. After the blood was coagulated, the experiment was repeated six times. Table 6 shows the average content of elements in cloth with and without bloodstains of 6 samples, and Table 7 shows that in swabs with and without bloodstains of six samples. Normality and homoscedasticity of data were assessed and a *t*-test was applied when comparing two groups. For the average content of Sn, Sb, Ba, and Pb, no significant differences were found between samples with and without bloodstains (*P* > 0.05).

### GSRs with bloodstain detection on cloth

The concentrations of Sn, Sb, Ba, and Pb in GSRs at the bullet hole were higher than those around the bullet hole. Furthermore, the concentration of Sn, Sb, Ba, and Pb in GSRs at the bullet hole were dependent on distance. Therefore, the concentrations of Sn, Sb, Ba, and Pb in GSRs around the incident bullet hole were used to investigate the different shooting distances. The ultrasonic vibration method was used to pretreat samples of target cloth at a distance of 1.5 cm from the centre of the bullet hole for shooting distances of 20 and 60 cm using type 92 pistol and type 95-1 rifle (Table 8).

Table 8 shows the concentrations of Sn, Sb, Ba, and Pb in GSRs on cloth with bloodstains. The concentration of Pb for type 95-1 rifle was higher than that of type 92 pistol. In addition, the concentration of Sn, Sb, and Ba for type 95-1 rifle was less than that for type 92 pistol. The content of the propellant in munitions for type 95-1 rifle was higher than that in munitions for type 92 pistol. Although the type 95-1 rifle exhibited a higher initial velocity (*V*_92_ = 360 m/s, *V*  _95–1_ = 920 m/s), Pb concentration for type 95-1 rifle was higher than for type 92 pistol because of difference in munitions. The density of Sn, Sb, and Ba (3.51 g/cm, 6.68 g/cm, 7.28 g/cm) was less than that of Pb (11.34 g/cm). Thus, the amount of Sn, Sb, and Ba in GSRs for type 92 pistol was lower than that of type 95-1 rifle.

**Table 8:** Concentrations (μg/L) of Sn, Sb, Ba, and Pb in gunshot residues (GSRs) on target cloth at different shooting distances using type 92 pistol and type 95-1 rifle by ultrasonic vibration method.

Samples	Concentrations (μg/L)			
	Sn	Sb	Ba	Pb
Type 92 pistol-20 cm				
NO.1	948.47	454.47	274.26	973.45
NO.2	894.70	524.36	229.63	1039.48
NO.3	954.24	598.22	258.71	1183.24
Type 92 pistol-60 cm				
NO.1	20.45	16.45	13.01	50.16
NO.2	15.05	12.60	4.84	44.94
NO.3	21.80	17.56	13.35	52.31
Type 95 pistol-20 cm				
NO.1	189.54	154.72	88.51	1545.15
NO.2	143.48	104.21	73.27	1302.31
NO.3	126.43	93.47	68.47	1103.95
Type 95 pistol-60 cm				
NO.1	59.34	78.58	60.12	312.05
NO.2	49.51	63.47	48.63	284.21
NO.3	42.75	59.83	36.46	293.90

**Table 9:** Concentrations (μg/L) of Sn, Sb, Ba, and Pb ingunshot residues (GSRs) on the hands with bloodstains.

Samples	Concentrations (μg/L)			
	Sn	Sb	Ba	Pb
Type 95-1 rifle				
Blank hand	3.18	1.33	25.81	21.98
Blank swab with bloodstains	2.71	<LOD	2.41	1.26
1 shot	491.82	134.25	156.22	311.65
1 shot and 1 washing	32.25	17.56	46.25	39.64
1 shot and 2 washing	21.52	15.26	39.24	30.25
1 shot and 3 washing	14.31	9.54	42.52	29.53
Type 92 pistol				
Blank hand	5.41	1.26	23.13	25.45
Blank swab with bloodstains	1.15	<LOD	1.89	1.56
1 shot	591.17	268.9	556.22	513.24
1 shot and 1 washing	34.62	37.83	42.45	40.23
1 shot and 2 washing	28.45	29.38	41.84	40.12
1 shot and 3 washing	12.85	17.52	23.51	26.1

### GSRs with bloodstain detection on hands

Table 9 indicates that the concentrations of Sn, Sb, Ba, and Pb in GSRs on the thumb and index finger of the shooter’s right hand for type 95-1 rifle were higher than that of type 92 pistol. For a large content of ammunition in firearms, the shooter displayed a high concentration of GSRs on his/her hand.

## Conclusions

ICP-MS is a powerful tool for the analysis of GSRs with bloodstains. Concentrations of Sn, Sb, Ba, and Pb in GSRs with bloodstains can be measured by ICP-MS. The analysis of GSR plays a very important role in the resolution of gun-related cases. As the number of shooting cases such as robbery and homicide increases, higher requirements have been put forward for the inspection of GSRs. GSR samples are usually characterised in a small quantity and a complex composition. Additionally, their detection is difficult. GSR samples contain rich evidence, as their composition and quantity are closely related to the type of guns, type of bullets, target objects, shooting distance, extraction time, and extraction method. Therefore, GSRs in different objects are treated differently.

ICP-MS can effectively solve the issues encountered by SEM–EDX analyses in detecting GSRs with bloodstains. Considering the interference of bloodstains on GSRs, microwave digestion and ultrasonic vibration were investigated. The best pretreatment method for incident bullet holes was microwave digestion. On the other hand, the best pretreatment method for the region around the bullet hole and the shooter’s hand was ultrasonic vibration.

GSRs with bloodstains on the thumb and index finger of the shooter were investigated for different shooting distances for type 92 pistol and type 95-1 rifle. For the same shooting distance, GSRs collected from the cloth and the shooter’s hand with type 95-1 rifle were higher than those of type 92 pistol, and the shooting residues were still detected for a longer shooting distance with type 95-1 rifle. However, GSRs were not detected after the shooter washed his/her hands once. The results show that GSRs with bloodstains could be successfully detected by ICP-MS and that the bloodstains did not interfere in GSRs analysis.
